# The Interplay of Exogenous Cannabinoid Use on Anandamide and 2-Arachidonoylglycerol in Anxiety: Results from a Quasi-Experimental Ad Libitum Study

**DOI:** 10.3390/ph17101335

**Published:** 2024-10-06

**Authors:** Renée Martin-Willett, Carillon J. Skrzynski, Ethan M. Taylor, Cristina Sempio, Jost Klawitter, L. Cinnamon Bidwell

**Affiliations:** 1 Department of Psychology & Neuroscience, University of Colorado Boulder, Boulder, CO 80309, USA; rema8106@colorado.edu (R.M.-W.); carillon.skrzynski@colorado.edu (C.J.S.); ethan.taylor@colorado.edu (E.M.T.); 2 Department of Anesthesiology, iC42 Clinical Research and Development, University of Colorado Anschutz Medical Campus, Aurora, CO 80045, USA; cristina.sempio@ucdenver.edu (C.S.); jost.klawitter@ucdenver.edu (J.K.); 3 Institute of Cognitive Science, University of Colorado Boulder, Boulder, CO 80309, USA

**Keywords:** THC, CBD, anxiety, AEA, 2-AG, cannabinoid content, endocannabinoid system

## Abstract

The public is increasingly reporting using cannabis for anxiety relief. Both cannabis use and the endocannabinoid system have been connected with anxiety relief/anxiolytic properties, but these relationships are complex, and the underlying mechanisms for them are unclear. **Background/Objectives**: Work is needed to understand how the endocannabinoid system, including the endocannabinoids anandamide (AEA) and 2-arachidonoylglycerol (2-AG), may be impacted by the main constituents of cannabis, Δ9-tetrahydrocannabinol (THC), and cannabidiol (CBD). **Methods**: The current study examined how the ab libitum use of products differing in THC and CBD affected AEA and 2-AG among 292 individuals randomly assigned to THC-dominant use (N = 92), CBD-dominant use (N = 97), THC + CBD use (N = 74), or non-use (N = 29). **Results:** The findings suggest that AEA levels do not change differently based on 4 weeks of cannabis use or by cannabinoid content, as AEA similarly increased across all conditions from study weeks 2 to 4. In contrast, AEA decreased at an acute administration session with product conditions containing any THC having greater AEA levels on average than the non-use condition. With regard to 2-AG, its levels appeared to primarily be affected by THC-dominant use, both acutely and over 4 weeks, when controlling for baseline cannabis use and examining study product use frequency among use conditions. **Conclusions:** Overall, the results continue to shed light on the complicated relationship between cannabinoid content and endocannabinoid production, and highlight the need for continued research on their interplay in human subjects.

## 1. Introduction

The United Nations estimated 218 million cannabis users globally in 2020 [1], and meta-analytic data suggest that anxiety is among the most common reasons individuals choose to self-medicate with cannabis [2]. However, much of the existing research on medical cannabis use is equivocal as to whether exogenous cannabinoids, such as Δ9-tetrahydrocannabinol (THC) and cannabidiol (CBD), have therapeutic effects [3]. Furthermore, there remains a great deal to be learned about the mechanisms by which exogenous cannabinoids act upon the body towards clarifying the anxiogenic or anxiolytic properties of these compounds. In this endeavor, a greater understanding of the endocannabinoid system is a crucial component.

The endocannabinoid system (ECS) is an important modulator of the central nervous system [4] composed of three components: endogenous, lipid-based endocannabinoid neurotransmitters, enzymes that degrade and synthesize endocannabinoids, and cannabinoid receptors (CB1 and CB2) [4,5]. THC, the most prominent psychoactive component in cannabis acts as a partial agonist on CB1 and CB2 [6], while CBD is believed to act upon CB1 and CB2 as a non-competitive negative allosteric modulator [7,8]. It is also believed that THC and CBD interact with endocannabinoid neurotransmitters, though these mechanisms are less understood.

The most studied endocannabinoids to date are anandamide (N-arachidonoyl ethanolamide; AEA) and 2-arachidonoylglycerol (2-AG) [9]. These two compounds have a similar chemical structure and short half-life but are distinct in important ways [10]. AEA is a partial agonist of CB1 and functions to regulate boundary lipid function in response to physiological stimuli that have been implicated in anxiety etiology [11]. It is derived mainly from the cleavage of N-arachidonoyl phosphatidylethanolamine (NAPE) [12] and degraded by intracellular fatty acid amide hydrolase (FAAH) [13] and N-acylethanolamine acid amidase (NAAA) enzymes [14]. Concentrations of AEA in the central nervous system are magnitudes lower than 2-AG [15], and it has a much lower efficacy for cannabinoid receptors (acting as a partial agonist) than 2-AG, which is a full agonist [16]. Additionally, in contrast with 2-AG, AEA can bind with vanilloid receptors [17].

2-AG has a synergistic pathway with AEA [18] and has also been implicated in the etiology of mood disorders such as anxiety and depression [19,20]. 2-AG is synthesized by the phospholipase C (PLC)-diacylglycerol (DAG) lipase pathway [21] or by the hydrolysis of inositol phospholipids [22]. It is degraded enzymatically, partially by FAAH, but primarily by monoacylglycerol lipase (MAGL) and diacylglycerol lipase (DAGL) [23].

As described by Nahas and colleagues [24], THC does not interact directly with AEA, but potentially through two indirect pathways. Specifically, THC is believed to dose-dependently decrease AEA concentrations at the synapse, either when binding to CB1 or by influencing membrane proteins permeating the lipid bilayer. Building on this early pre-clinical work, research in humans is accumulating to support this. For example, one early study demonstrated that THC administration was related to a decrease in AEA concentrations within hours [25], while another very recent study found THC administration was related to decreases in AEA plasma concentration, with progressively lower pre-administration AEA levels between each of four trials, occurring a median of 14 days apart [26]. However, the timing of THC administration and AEA measurement may be critical in clarifying this relationship, as Thieme and colleagues reported a biphasic effect in which AEA initially increased from baseline levels but then fell again after 48 h [17].

While we know that CBD antagonizes the effects of THC at the receptor site [27], we know less about the mechanisms by which CBD acts on AEA. Currently proposed mechanisms support the hypothesis that CBD has an opposite effect on AEA compared to THC and increases AEA concentrations [28]. These possible mechanisms include the inhibition of lipoxygenases involved in AEA degradation, competitive binding to proteins that transport AEA towards FAAH enzymes for catabolism, or mediating the agonism bias of CB1 and CB2, which in turn changes the effect of AEA, though the experimental component of the study cited here found no significant effect of CBD on AEA over a period of 28 days [28]. There is less research on the relationship between CBD and AEA, and pre-clinical results have been mixed or do not specifically measure AEA concentrations following CBD administration, but rather inflammatory processes thought to be related to AEA [29]. The results are also equivocal in humans. For example, in the recent study described above, CBD had no effect on AEA concentrations [26], while other earlier work has reported that CBD increased AEA concentrations in schizophrenia [30].

Less is known about the mechanisms by which THC and CBD act on 2-AG. In Thieme’s study, 2-AG levels mirrored the biphasic changes in AEA after THC administration [17], while Maia and colleagues found that 2-AG levels in human tissue were not affected by THC administration, even though MAGL and DAGL were significantly impaired [31]. One administration study found that low-potency THC did not have an effect on 2-AG levels acutely (15–180 min after administration), but reported that 2-AG was significantly, negatively associated with frequency of THC use and blood levels of THC prior to the experiment [32], once again suggesting differential effects of the short- vs. longer-term use of THC on endocannabinoids.

In the same way that CBD negatively modulates the effects of THC at CB1, so it is believed to act upon 2-AG [8,33], though we know even less about this relationship compared to others described here. Preclinical work has been varied, with studies such as that of Remiszewski et al. reporting decreased 2-AG, versus Baranowksa-Kuczko et al., who reported increased 2-AG levels, both after 2 weeks of CBD administration in a rodent model [34,35]. There is much less work in humans on CBD and 2-AG, though Chester et al. recently reported that CBD administration had no effect on 2-AG levels at timepoints roughly 14 days after administration [26]. The field is sorely in need of additional data in humans on this relationship.

In summary, indications in the literature that AEA and 2-AG concentrations may play a prominent role in the symptomology of anxiety, and increasing evidence that they are modulated by exogenous cannabinoid use, suggests that further understanding of the relationships between AEA and 2-AG, THC and CBD, and their effects on anxiety would be highly beneficial to moving the field forward in this area. Thus, our primary research question presented in this study was whether the ad libitum use of legal market cannabis with differing cannabinoid contents was related to changes in AEA and 2-AG. Given the literature’s suggestion that short- and longer-term effects may differ, we examined these associations both under the acute influence of cannabinoids and over a 4-week period of naturalistic exposure.

## 2. Results

### 2.1. Participants and Study Design

This study utilized data from a larger study described elsewhere [36]. The current sample included 292 individuals who had available endocannabinoid data. Participants in the present analyses (59.9% assigned female at birth, 38.7% assigned male at birth, 1.37% unknown) reported a mean age of 33.30 ± 13.56 years and had mild anxiety or greater (scores ≥ 5 on the GAD-7 scale [17]). Additional sample characteristics are provided in Table 1. The participants were randomly assigned to one of four conditions, including both flower and edible products: a THC-dominant product (flower products: 24% THC, <1% CBD; edible products: 10 mg THC, 0 mg CBD; N = 92), a CBD-dominant product (flower products: <1% THC, 24% CBD; edible products: 0.17 mg THC, 10 mg CBD; N = 97), a 1:1 formulation of THC and CBD (flower products: 12% THC, 12% CBD; edible products: 10 mg THC, 10 mg CBD; N = 74), and a control group not using cannabis (N = 29). This study includes two sets of analyses. First, in the extended models, the participants used their products ad libitum following a baseline appointment for 2 weeks, at which time they attended a study visit at the laboratory. This was followed by another 2 weeks of use that culminated at a mobile pharmacology laboratory visit at their homes. We selected a 30-day period to maximize the experimental period while balancing feasibility, given that the previous literature is lacking in studies over longer time periods, and the existing research is equivocal [36]. For the second, acute-administration models, the participants provided pre-use measures in the mobile pharmacology lab prior to use and then again after use. The time between assessments for flower users averaged 17 min, while time between assessments for edible users was approximately 60 min. This difference in time was to ensure that the participants had consistent cannabinoid exposure during the acute session (i.e., accounting for the difference in time it takes for the body to metabolize inhaled versus edible cannabis) [37]. Correlations between the levels of AEA and 2-AG between each timepoint are detailed in Table 2. Of note, the differentiating effects between administration methods (edible versus inhaled) were beyond the scope of these analyses.

### 2.2. Manipulation Check Comparing Blood Levels of CBD and THC across Conditions

**Figure 1:**
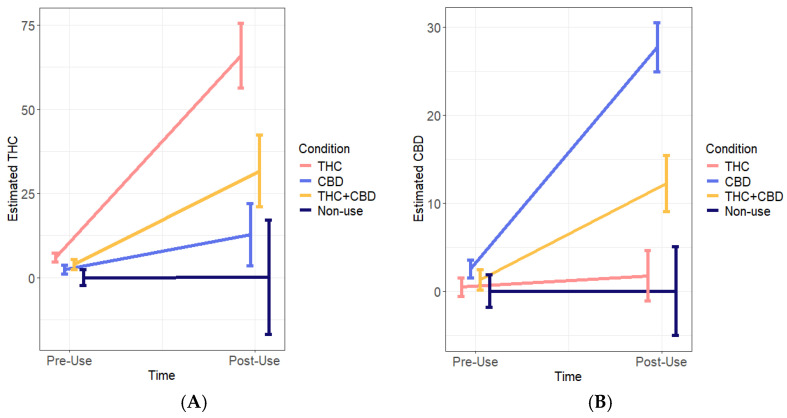
Blood THC (**A**) and CBD (**B**) across conditions from pre- to post-use. Blood was collected at both timepoints (pre- and post-cannabis use for cannabis-use conditions) during van visit. THC N = 92, CBD N = 97, THC + CBD N = 74, non-use N = 29. Bars represent standard errors.

Blood levels of THC differed at both pre-use and post-use timepoints across the conditions, such that THC levels were higher among the THC condition compared to the CBD and non-use conditions (ps ≤ 0.05) at pre- and post-use. At post-use, THC was also higher in this condition than the THC + CBD condition (*p* = 0.02). THC also significantly increased from pre- to post-use for both the THC and THC + CBD conditions (995.183% and 720.82% changes, respectively; ps < 0.01). In contrast, CBD levels were not different at pre-use across the conditions. However, CBD levels did significantly increase from pre- to post-use for both the CBD and THC + CBD conditions (993.50% and 870.86% changes, respectively; ps < 0.001), and were significantly higher at post-use within the CBD condition compared to all three conditions (ps < 0.001). Likewise, CBD was higher in the THC + CBD condition at post-use compared to the THC and non-use conditions (ps < 0.05). See Figure 1A,B for changes in pre- to post-use THC and CBD blood across conditions, respectively.

### 2.3. Comparing THC, CBD, THC + CBD, and Non-Use Conditions on Extended and Acute AEA and 2-AG Changes

#### 2.3.1. Extended AEA Changes: AEA Levels Increased from 2 to 4 Weeks but Do Not Differ across THC, CBD, THC + CBD, or Non-Use Conditions

**Figure 2:**
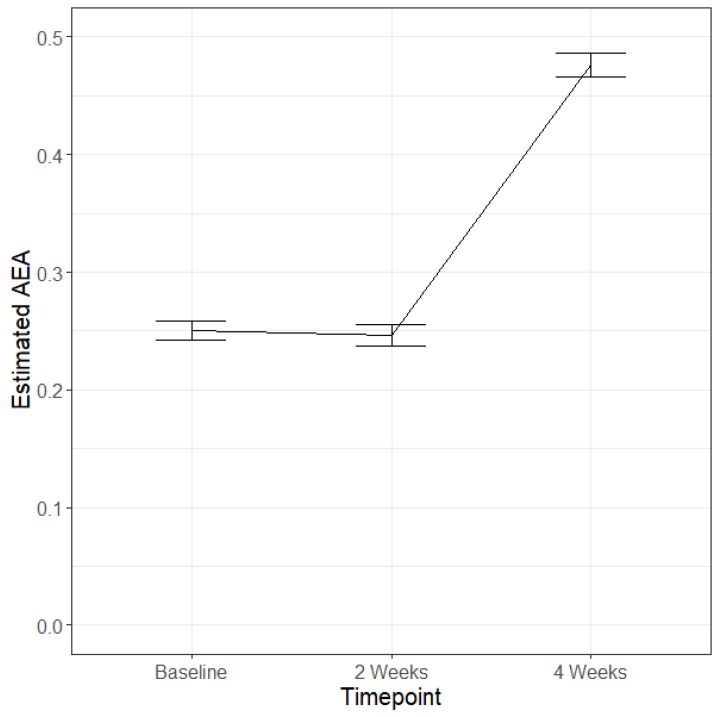
Estimated blood AEA levels over the four weeks of this study on average across the four conditions. Blood was collected at all timepoints; baseline, 2 weeks, and 4 weeks. THC N = 92, CBD N = 97, THC + CBD N = 74, non-use N = 29. Bars represent standard errors.

AEA did not change over the 4 weeks differently based on product use; instead, on average across conditions, there was no change in AEA levels from baseline to 2 weeks, while from 2 weeks to 4 weeks, AEA levels increased (48.34% change; see Table 3 and Figure 2).

#### 2.3.2. Extended 2-AG Changes: 2-AG Levels Did Not Change over 4 Weeks across the THC, CBD, THC + CBD, and Non-Use Conditions

There were no significant results regarding 2-AG changes across conditions over the 4 weeks of this study (see Table 1).

#### 2.3.3. Acute AEA Changes: AEA Levels Decreased after Immediate Use across All Conditions, and on Average across Van Session Timepoints, Differed between the THC and Non-Use Condition

**Figure 3:**
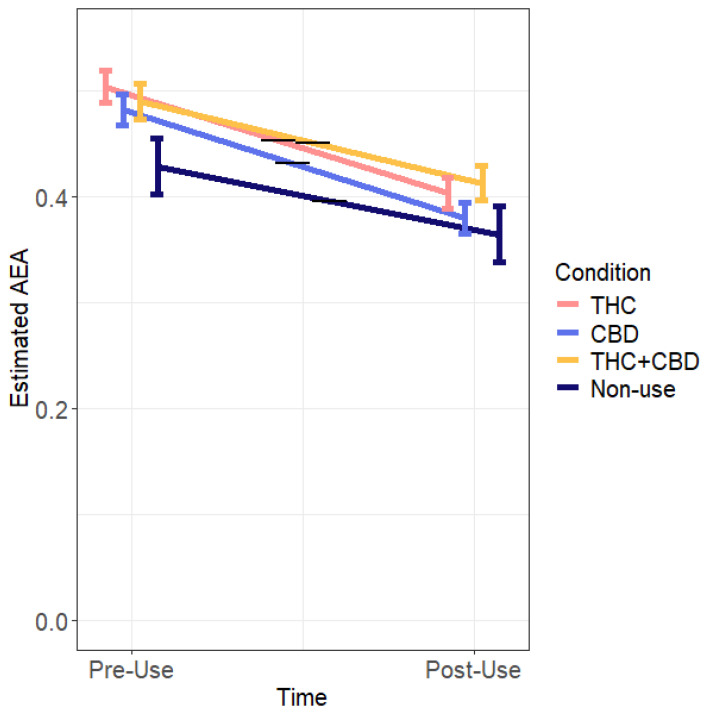
Estimated AEA levels over acute timepoints across conditions. Blood was collected at both timepoints (pre- and post-cannabis use for cannabis-use conditions) during van visit. THC N = 92, CBD N = 97, THC + CBD N = 74, non-use N = 29. Bars represent standard errors. Note. Black bars denote estimated AEA averages over time.

AEA decreased from pre- to post-use (18.05% change), but this decrease was not different across conditions. However, on average across time, the THC condition had higher AEA levels than the non-use condition, and the THC + CBD condition had marginally higher AEA levels than the non-use condition (see Table 1 and Figure 3).

#### 2.3.4. Acute 2-AG Changes: 2-AG Levels Did Not Change after Immediate Use across the THC, CBD, THC + CBD, and Non-Use Conditions

2-AG did not change differently across conditions after immediate cannabis use (see Table 1).

### 2.4. Comparing THC, CBD, and THC + CBD Conditions on Extended AEA Changes, Controlling for Baseline Cananbis Use and Including Moderation by Study Product Use

Comparisons between the three cannabis conditions over the 4 weeks of use were additionally explored, including the baseline frequency of cannabis use (i.e., total days of cannabis use across the 30 days prior to the start of this study) as a covariate, and study product use frequency (i.e., total days of study product use across the 4 weeks of this study) as a moderator.

#### 2.4.1. Extended AEA Changes: AEA Levels Increased over 4 Weeks across All Three Use Conditions

As in previous findings that included the non-use condition, AEA increased from 2 to 4 weeks (97.59% change); however, this increase did not differ between conditions and did not depend on study product use frequency (see Table 4). No other results were significant.

#### 2.4.2. Extended 2-AG Changes: 2-AG Levels Changed over 4 Weeks for the THC Condition

**Figure 4:**
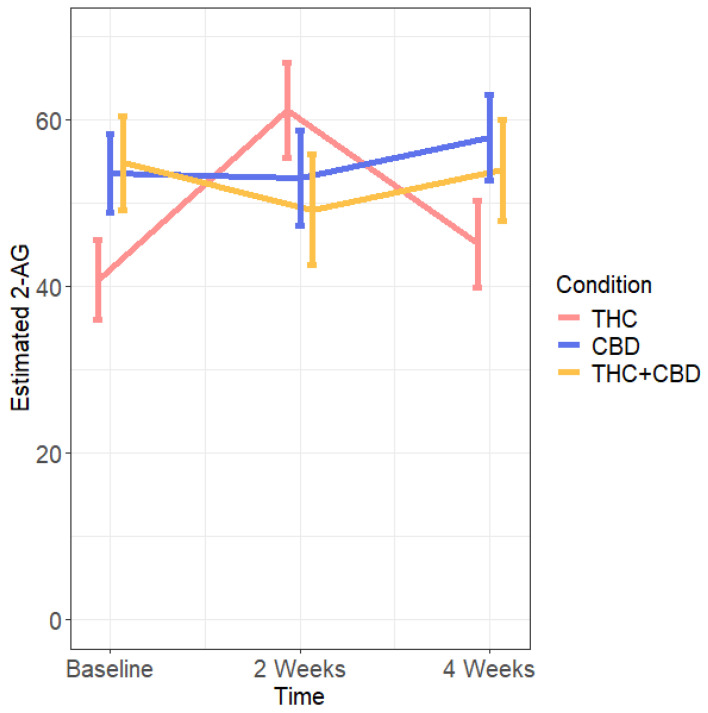
Estimated 2-AG levels over time across use conditions. Blood was collected at all timepoints; baseline, 2 weeks, and 4 weeks. THC N = 92, CBD N = 97, THC + CBD N = 74, non-use N = 29. Bars represent standard errors.

2-AG did change differently over time based on the conditions. Specifically, 2-AG levels were marginally lower within the THC condition compared to both the CBD and THC + CBD condition at baseline, and marginally lower than the CBD condition at 4 weeks (see Table 2). Further, the CBD and THC + CBD conditions did not change over the 4 weeks, but the THC condition increased from baseline to 2 weeks (50.17% change), and then decreased from 2 weeks to 4 weeks (26.25% change), returning to baseline levels (i.e., no difference between baseline and week-4 2-AG levels) (see Table 2 and Figure 4).

## 3. Discussion

This study is one of the first to examine how the immediate and 4-week ab libitum use of products differing in THC and CBD affects AEA and 2-AG. Additionally, no other study to our knowledge has examined these relationships over the course of 4 weeks in humans, which is a significant contribution to the growing endocannabinoid literature. Other strengths of the current study include the use of legal market products with “real world” THC and CBD potencies commonly used by the public, as well as random assignment to products containing different ratios of THC to CBD. This allowed for greater external validity (i.e., individuals were using products that are widely available) while still aiming to balance experimental verification given the legal constraints on cannabis research [38,39].

The findings indicated that AEA increased similarly across conditions from 2 to 4 weeks and decreased from pre- to post-use acutely across conditions. However, on average, at the acute administration session, the THC and THC + CBD conditions had higher AEA levels than the non-use condition. These results are consistent with the small body of prior research on the short-term effects of THC [25,26], and may support the hypothesis that the effects of THC on AEA are driven by competitive binding between the two compounds at CB1 [16]. The finding that 2-AG was not affected by cannabinoid content when all four conditions were included in models is also consistent with the competitive binding hypothesis. This could perhaps be due to 2-AG’s full agonist action at CB1, as well as considerably higher concentration levels overall (compared to AEA), which could cause changes in concentrations to be less significant [16]. These results align with Hua and colleague’s study that reported no significant changes in AEA after CBD administration [28], but are in contrast to Leweke et al.’s report of increased AEA [30]. This may be related to the populations studied, however, as Hua’s study was among participants with cannabis use disorder, while Leweke’s was among participants with a schizophrenia diagnosis. More mechanistically focused research is needed given the potential significance of the etiology of mood disorders [11,19,20].

In contrast, a significant biphasic pattern in concentration change over time emerged for 2-AG in the THC condition, but only when models excluded the non-use group. These results were similar to both Kearney-Ramos et al.’s [32] and Thieme and colleagues’ studies [17] that were 14 days and 2 days long, respectively. Taken together with Chester et al.’s recent findings of progressively lower baseline endocannabinoid levels between administrations, it may be possible that THC has immediate and cumulative effects that result in oscillations of decreasing magnitude of endocannabinoid concentration. However, this hypothesis is highly speculative and should also be considered in light of the findings from Maia et al. looking at endocannabinoids in placental tissue. In this case, 2-AG-involved enzymatic activity was more significantly impacted by THC than 2-AG itself [31]. Future work is again needed across longer time periods with more frequent measurements to test this.

Finally, our study found minimal effects of CBD on endocannabinoid levels. Our results largely replicate findings from the one comparable prior study in humans, but any hypotheses as to what is driving these results would be highly speculative [26]. Some recent evidence suggests that the effects of CBD can vary significantly depending on dose, though pharmacokinetic research is lacking [40,41]. Other recent studies even suggest that very high doses of CBD can increase, and not mitigate, the effects of THC [37]. Thus, our observed effects could perhaps be due to low doses of CBD in our study. Again, more research is needed among humans to shed further light on these relationships.

Our study is not without limitations that should be noted. Despite the random assignment of products to study participants, this was not a fully blinded and dose-controlled trial. As the legal landscape continues to evolve, the field would surely benefit from RCTs using products that are equivalent to products used by the public in the “real world”. Additionally, we did not account for the potential differences in effects caused by administration method. Some of our own data have suggested that cannabis form may have differential effects on outcomes like sleep [42], and thus our future work will explore this question for endocannabinoid functioning.

## 4. Materials and Methods

This research sample is derived from the study “Novel Approaches to Understanding the Role of Inflammation in Anxiety” (see funding statement). The full protocol was preregistered on clinicaltrials.gov. Detailed procedures for the larger study have been described previously [36], and thus shall be provided in brief here.

### 4.1. Recruitment, Timeline, and Compensation

Participants were recruited through postal mail, social media, and community outreach in the Denver–Boulder metro area starting in March 2017 through to December 2022. The inclusion criteria included that individuals must have been experiencing mild to moderate levels of anxiety and intended to initiate cannabis use for anxiety symptoms. Individuals who would use cannabis in the study all had at least one lifetime episode of cannabis use, while those not using cannabis had not used cannabis for the previous 6 months. This project followed all ethical standards and the Helsinki Declaration of 1975 as revised in 2008, and was approved by the University of Colorado Boulder Institutional Review Board.

### 4.2. Measures

Demographics such as gender and age were gathered at baseline. The participants reported their cannabis use at baseline, 2-week, and 4-week study visits using an online version of Timeline Followback (O-TLFB) [43,44,45]. Their blood cannabinoid levels were also assessed. Blood was collected in a 7 mL glass EDTA tube for endocannabinoid quantitation by a certified phlebotomist at each timepoint. At the 4-week mobile appointment, blood was stored on ice until returning to the campus laboratory. These samples were centrifuged at 1000× *g* for 10 min to separate erythrocytes from plasma. In total, 900 μ of this plasma was mixed with 100 μL of a 5% formic acid solution and stored at −80 °C until analysis at the iC42 Laboratory at the University of Colorado Anschutz School of Medicine. Fourteen endocannabinoids were quantified using liquid chromatography tandem mass spectrometry (LC/LC-APCI-MS/MS) at the iC42 Laboratory. This method was validated, as well as being inter- and intra-run for accuracy and precision at iC42 [46].

### 4.3. Data Analysis

Preliminary analyses testing baseline differences across product conditions have been analyzed and published in a previous article [36], and indicated successful randomization (i.e., product conditions did not differ on demographic or cannabis use variables). Primary analyses to investigate if 2-AG and AEA differed by product condition over time followed analyses from this prior paper and included separate mixed-effect models (MLMs) using the nlme package in R [47] to examine extended time changes and analysis of variance (ANOVA) tests for acute changes. Specifically, models were run with each endocannabinoid as a separate outcome and included as predictors the condition, time (baseline, 2-week, and 4-week timepoints for extended models versus pre-use and post-use timepoints for acute models), and the interaction between condition and time. A MLM output including *p*-values was generated using the anova.lme function, while the summary function generated *p*-values for ANOVA tests.

Another set of MLMs was additionally run to examine extended effects on the endocannabinoids, but including only the use conditions. In this case, the baseline frequency of cannabis use (i.e., total days of cannabis use across the 30 days prior to the start of this study) was included as a covariate. We also included study product use frequency (i.e., total days of study product use across the 4 weeks of this study) as a moderator of the relationship between condition and endocannabinoid change. Where significant interactions existed, post hoc analyses were run using the emmeans function [48], which produces estimated marginal means and *p*-values for specified points of interest (e.g., estimated THC blood levels for THC vs. CBD conditions at the post-use timepoint) by a weighted regression method. Graphs of these estimated outcomes based on model findings were also generated to help with visualization using the ggplot2 function [49]. Across all models, random intercepts and slopes were included (i.e., intercepts and slopes were allowed to vary per person), and maximum likelihood estimation was utilized to account for missingness over time. All analyses were conducted using R [50].

## Figures and Tables

**Table 1:** Participant characteristics across conditions.

	Non-Users (n = 29)	THC + CBD (n = 74)	CBD (n = 97)	THC (n = 92)
Baseline Participant Characteristics				
Age (Mean (SD))	35.28 (14.71)	34.47 (14.34)	32.09 (13.16)	32.99 (13.03)
Sex Assigned at Birth (No. (%) Female)	62.07	58.11	57.73	63.04
Education (No. (%) Bachelors or Higher) *	86.21	56.76	57.73	59.78
Employment (No. (%) Full Time Employed)	51.72	37.84	46.39	45.65
Race (%)				
American Indian or Alaska Native	6.90	1.35	5.15	6.52
Black or African American	3.45	2.70	3.09	3.26
Hispanic or Latino	13.79	5.41	7.22	5.43
More Than One Race/Ethnicity	0.00	2.70	0.00	2.17
Native Hawaiian or Other Pacific Islander	0.00	0.00	0.00	1.09
Prefer not to answer	3.45	2.7	3.09	1.09
White	68.97	81.08	80.41	78.26
Baseline Psychological Functioning (Mean(SD))				
DASS Scoring: Depression	13.31 (9.12)	13.49 (9.6)	14.82 (9.76)	14.24 (9.41)
DASS Scoring: Anxiety	8.97 (7.68)	9.78 (6.42)	10 (7.83)	9.26 (6.36)
DASS Scoring: Stress	19.45 (9.71)	18.32 (8.9)	18.82 (8.19)	17.17 (7.73)
Baseline Cannabis Characteristics (Mean(SD))				
Days of Flower Use (past 14 days) ***	0.00 (0.00)	4.33 (5.28)	3.38 (4.71)	3.24 (4.34)
Days of Edible Use (past 14 days)	0.00 (0.00)	1.19 (2.61)	1.11 (2.43)	0.79 (1.75)
Days of Cannabis Use (past 14 days) ***	0.00 (0.00)	5.85 (5.39)	5.57 (5.07)	5.18 (4.90)
Cannabis Use Disorder Symptoms (MDS) ***	0.00 (0.00)	3.21 (4.19)	2.91 (3.83)	2.65 (3.63)
Study Cannabis Use (Mean (SD))				
Study Assigned Product Use	0.00 (0)	2.30 (7.89)	0.58 (8.09)	1.49 (8.7)
Baseline Endocannabinoid Levels (ng/mL)				
AEA	0.22 (0.12)	0.28 (0.15)	0.25 (0.13)	0.25 (0.12)
2-AG	52.46 (37.29)	51.5 (47.18)	53.85 (48.38)	40.27 (37.09)

Note. * indicates *p* < 0.05; *** indicates *p* < 0.001.

**Table 2:** Correlations between endocannabinoid levels across all timepoints.

	T00 AEA	T00 2-AG	T01 AEA	T01 2-AG	Ta2 AEA	Ta2 2-AG	Tb2 AEA
T00 AEA							
T00 2-AG	0.029 (0.626)						
T01 AEA	0.365 (<0.001)	−0.024 (0.699)					
T01 2-AG	−0.020 (0.745)	0.273 (<0.001)	0.064 (0.290)				
Ta2 AEA	0.208 (<0.001)	−0.107 (0.067)	0.260 (<0.001)	−0.059 (0.330)			
Ta2 2-AG	−0.076 (0.195)	0.255 (<0.001)	−0.067 (0.270)	0.315 (<0.001)	−0.037 (0.534)		
Tb2 AEA	0.284 (<0.001)	−0.044 (0.457)	0.254 (<0.001)	−0.001 (0.989)	0.623 (<0.001)	−0.070 (0.230)	
Tb2 2-AG	0.029 (0.625)	0.184 (0.002)	−0.018 (0.770)	0.159 (0.009)	−0.005 (0.928)	0.371 (<0.001)	0.049 (0.408)

Note. Computed correlation used Pearson method with pairwise deletion. T00 = baseline timepoint; T01 = 2-week timepoint; Ta2 = 4-week, pre-acute use timepoint; Tb2= 4-week, post-acute use timepoint.

**Table 3:** Mixed-effect models assessing AEA and 2-AG over all four conditions across four weeks (i.e., extended time) and immediate use (i.e., acute time).

	AEA			2-AG		
Predictors	*F*	Part *η*^2^	*p*-Value	*F*	Part *η*^2^	*p*-Value
Extended models						
Extended Time	298.17	0.52	<0.001	1.11	0.00	0.33
Condition	1.40	0.01	0.24	1.27	0.01	0.29
Extended Time × Condition	1.17	0.01	0.32	1.75	0.02	0.11
Acute models						
Acute Time	60.19	0.09	<0.001	1.62	0.00	0.20
Condition	2.93	0.02	0.03	0.74	0.00	0.53
Acute Time × Condition	0.47	0.00	0.70	0.91	0.00	0.44

**Table 4:** Mixed-effect models assessing AEA and 2-AG over the three use conditions across four weeks (i.e., extended time), controlling for baseline use frequency and examining study product use frequency moderation.

	AEA			2-AG		
Predictors	*F*	Part *η*^2^	*p*-Value	*F*	Part *η*^2^	*p*-Value
Extended Time	254.26	0.52	<0.001	1.13	0.00	0.32
Condition	0.57	0.00	0.56	1.31	0.01	0.27
Baseline Use frequency	0.55	0.00	0.46	1.47	0.00	0.23
Study Use frequency	0.65	0.00	0.42	0.00	0.00	0.95
Extended Time × Condition	0.85	0.00	0.49	2.51	0.02	0.04
Condition × Study Use Frequency	0.90	0.00	0.41	0.97	0.00	0.38

## Data Availability

The parent study for these analyses was pre-registered on clinicaltrials.gov (NCT03491384). The data presented in this study are available on request from the corresponding author due to the sensitive nature of the data (substance use) for participants.
